# Contractility, ventriculoarterial coupling, and stroke work after acute myocardial infarction using CMR‐derived pressure‐volume loop data

**DOI:** 10.1002/clc.24216

**Published:** 2024-01-16

**Authors:** David Nordlund, Theodor Lav, Robert Jablonowski, Ardavan Khoshnood, Ulf Ekelund, Dan Atar, David Erlinge, Henrik Engblom, Håkan Arheden

**Affiliations:** ^1^ Department of Clinical Sciences Lund, Clinical Physiology Lund University and Skane University Hospital Lund Sweden; ^2^ Department of Clinical Sciences Malmö, Emergency Medicine, Lund University Skane University Hospital Malmö Sweden; ^3^ Department of Clinical Sciences Lund, Emergency Medicine Lund University and Skane University Hospital Lund Sweden; ^4^ Dept. of Cardiology Oslo University Hospital Ulleval Oslo Norway; ^5^ Institute of Clinical Medicine University of Oslo Oslo Norway; ^6^ Cardiology, Department of Clinical Sciences Lund Lund University and Skane University Hospital Lund Sweden

**Keywords:** contractility, coupling, efficiency, elastance, myocardium at risk, stroke work

## Abstract

**Background:**

Noninvasive left ventricular (LV) pressure‐volume (PV) loops derived by cardiac magnetic resonance (CMR) have recently been shown to enable characterization of cardiac hemodynamics. Thus, such PV loops could potentially provide additional diagnostic information such as contractility, arterial elastance (E_a_) and stroke work (SW) currently not available in clinical routine. This study sought to investigate to what extent PV‐loop variables derived with a novel noninvasive method can provide incremental physiological information over cardiac dimensions and blood pressure in patients with acute myocardial infarction (MI).

**Methods:**

A total of 100 patients with acute MI and 75 controls were included in the study. All patients underwent CMR 2−6 days after MI including assessment of myocardium at risk (MaR) and infarct size (IS). Noninvasive PV loops were generated from CMR derived LV volumes and brachial blood pressure measurements. The following variables were quantified: Maximal elastance (E_max_) reflecting contractility, E_a_, ventriculoarterial coupling (E_a_/E_max_), SW, potential energy, external power, energy per ejected volume, and efficiency.

**Results:**

All PV‐loop variables were significantly different in MI patients compared to healthy volunteers, including contractility (E_max_: 1.34 ± 0.48 versus 1.50 ± 0.41 mmHg/mL, *p* = .024), ventriculoarterial coupling (E_a_/E_max_: 1.27 ± 0.61 versus 0.73 ± 0.17, *p* < .001) and SW (0.96 ± 0.32 versus 1.38 ± 0.32 J, *p* < .001). These variables correlated to both MaR and IS (E_max_: *r* ^2^ = 0.25 and *r* ^2^ = 0.29; E_a_/E_max_: *r* ^2^ = 0.36 and *r* ^2^ = 0.41; SW: *r* ^2^ = 0.21 and *r* ^2^ = 0.25).

**Conclusions:**

Noninvasive PV‐loops provide physiological information beyond conventional diagnostic variables, such as ejection fraction, early after MI, including measures of contractility, ventriculoarterial coupling, and SW.

## Graphical abstract

Contractility, ventriculoarterial coupling, and SW in healthy volunteers and myocardial infarction (MI) patients using noninvasive pressure‐volume (PV) loop measurements. The top box illustrates generation of PV loops non‐invasively by sphygmomanometry and CMR. Variables such as E_max_ (used as a measure of contractility), ventriculoarterial coupling, and SW were calculated. The left graph shows an example of a PV loop in a healthy volunteer where E_a_ and E_max_ have been illustrated as the slope of the dotted lines and SW as the gray area filling the PV loop. The right graph shows an example of a PV loop in a patient during the first week after MI and is representative of the mean difference in Emax (lower), VAC (higher), and SW (lower) compared to the healthy volunteers. CMR, cardiovascular magnetic resonance, E_a_, arterial elastance; E_max_, maximal elastance; PV, pressure volume; SW, stroke work; VAC, ventriculoarterial coupling. Image of MRI scanner by macrovector on Freepik.com. Image of sphygmomanometer cuff by www.medisave.co.uk

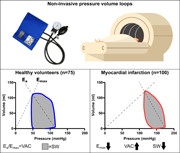

**Abbreviations**
CMR: cardiovascular magnetic resonance
E_a_
: arterial elastance
E_max_
: maximal elastance
MaR: myocardium at risk
MI: myocardial infarction
PE: potential energy
PV: pressure‐volume
SW: stroke work

## 1 INTRODUCTION

Ischemic heart disease is the most common cause of heart failure, currently considered one of the major healthcare challenges of our time.^1^ Despite the existence of effective treatments for heart failure, its prognosis remains poor and recent guidelines suggest a need for better measurements to target therapies and individualize treatments.^2^ Since the pathological basis of heart failure is complex, diagnostic methods need to enable assessment of myocardial characteristics beyond currently established diagnostic variables such as cardiac dimensions and ejection fraction. Heart failure can exist with both normal and abnormal cardiac dimensions and ejection fraction due to alterations in properties such as myocardial stiffness and contractility affecting the efficiency of cardiac work.^2^ These aspects of cardiac pathophysiology can be assessed from pressure‐volume (PV) loops yielding variables such as contractility, arterial elastance (E_a_), ventriculoarterial coupling, SW, potential energy (PE), external power, energy per ejected volume, and efficiency.

The use of PV loops, first described by Otto Frank in the late eighteen‐hundreds,^3^ has until now been predominantly as a research tool to selected patient populations because of the need for invasive procedures for their acquisition. Recently, a novel noninvasive method for calculating PV loops of the left ventricle (LV) was described and validated.^4^ This method can be used to assess cardiac mechanics and energetics by combining pressures derived from brachial sphygmomanometry with cardiac volumes from cardiac magnetic resonance (CMR) images.^3^, ^5^ Thus, PV loops can now be acquired in clinical routine in all patients undergoing CMR which allows assessment of hemodynamics in much larger patient populations.^4^, ^6^ Recent experimental data, using the new noninvasive method, explores the use of PV loops to elucidate pathophysiology after MI but to what extent PV‐loop variables derived from this method can provide incremental value over cardiac dimensions and blood pressure in patients with acute MI is unclear.^7^

The aim of this study was to investigate whether cardiac hemodynamics early after acute MI using data from noninvasive PV loops provides incremental diagnostic information over standard measurements, and to investigate to what extent this differs from normal hemodynamics.

## 2 METHOD

### 2.1 Study population

A total of 100 patients with acute myocardial infarction (MI) were included and underwent CMR at Lund University Hospital at 4 ± 2 days after acute percutaneous coronary intervention due to ST‐elevation MI (STEMI). The patients were included as part of three clinical trials: the SOCCER (*n* = 83), MITOCARE (*n* = 6), and CHILL‐MI (*n* = 11) trials.^8^, ^9^, ^10^ Inclusion and exclusion criteria are described in the Supporting Information: Table 1. In short, patients had symptom duration <6 h, no previous MI and no contraindications to CMR scanning. Patients with specified conditions such as congestive heart failure, hepatic failure, cardiac arrest were excluded. In addition, 75 age‐ and sex‐matched healthy volunteers from a local research database were retrospectively included in the study. The controls had no history of cardiovascular disease, were not regularly taking any medication and were non‐smokers. Data on individual medications was not available in the acute setting. In the period of the clinical trials, however, >90% of these patients received a beta‐blocker, an ACEi or ARB, a statin, and dual antiplatelet therapy. The CHILL‐MI, MITOCARE and SOCCER trials were approved by the local/regional institutional review boards/ethics committees. The study of healthy volunteers was approved by the regional ethical review board in Lund, Sweden. All study participants provided written consent. The study conforms to the principles outlined in the Declaration of Helsinki.

### 2.2 CMR image acquisition

All CMR examinations were performed on a 1.5 T system (Philips Healthcare, Best, The Netherlands). All subjects were placed in supine position and images were acquired at end‐expiratory breath hold with ECG gating. Scout images were acquired to locate the heart. For time‐resolved cine imaging of myocardial function, a multi‐slice multiphase steady state free precession (SSFP) sequence was applied approximately 5 min after intravenous administration of a gadolinium‐based extracellular contrast agent (0.2 mmol/kg). The contrast enhanced SSFP (CE‐SSFP) images (temporal resolution: 30 frames/cardiac cycle) were acquired in the short‐axis view (slice thickness: 8 mm; in‐plane resolution: typically 1.5 × 1.5 mm) covering the LV from base to apex and in 2‐, 3‐, and 4‐chamber views.

End‐diastolic short‐ and long‐axis late gadolinium enhancement (LGE) images in image planes corresponding to those for contrast enhanced SSFP were acquired at expiratory breath hold approximately 15 min after injection of contrast administration. The LGE‐images were acquired using an inversion‐recovery gradient‐recalled echo sequence (slice thickness: 8 mm, no slice gap; In‐plane resolution: typically 1.5 × 1.5 mm; inversion time adjusted to null viable myocardium).^11^

### 2.3 CMR image analysis

All CMR images were analyzed using the software Segment, version 3.3 (http://segment.heiberg.se).^12^

#### 2.3.1 PV loops

Volumetric data for the PV loops were acquired by evaluation of the time‐resolved cine images according to a previously described method.^4^ In short, the LV lumen was manually delineated in end‐diastole, end‐systole, and in one timeframe in diastasis. An automatic spline interpolation method was then used to fill out the remaining timeframes and achieve the LV volume variation over time throughout the cardiac cycle. This semi‐automatic method for volumetric analysis has recently been validated in healthy controls and heart failure patients.^13^ To ensure that the method is applicable also in MI patients with regional myocardial dysfunction, a subset of patients (*n* = 30) was evaluated using the same method but with manual corrections for akinesia/dyskinesia for comparison with the semi‐automatic method.

The method for generating PV loops has previously been described.^4^ It is based upon assumptions of a constant shape of time‐varying elastance and uses input of noninvasive blood pressure data and volumes from CMR imaging to calculate pressure throughout the cardiac cycle. The end diastolic pressure was set to 5 mmHg for the entire study population as per the original publication.^4^ The range is thought to represent the majority of the study population of which no one suffered severe heart failure, considering that most patients with heart failure maintain a LV end‐diastolic pressure of 5−20 mmHg, and considering that differences in LV diastolic pressures, within reasonable ranges, have little effect on the calculated PV‐loop parameters.^14^, ^15^ Brachial sphygmomanometry data was gathered from the CHILL‐MI study result sheets, from the healthy control data sheets, and from clinical patient charts (SOCCER and MITOCARE) in accordance with informed consent. All blood pressures were taken at rest and on the same day as the CMR examination.

**Figure 1:**
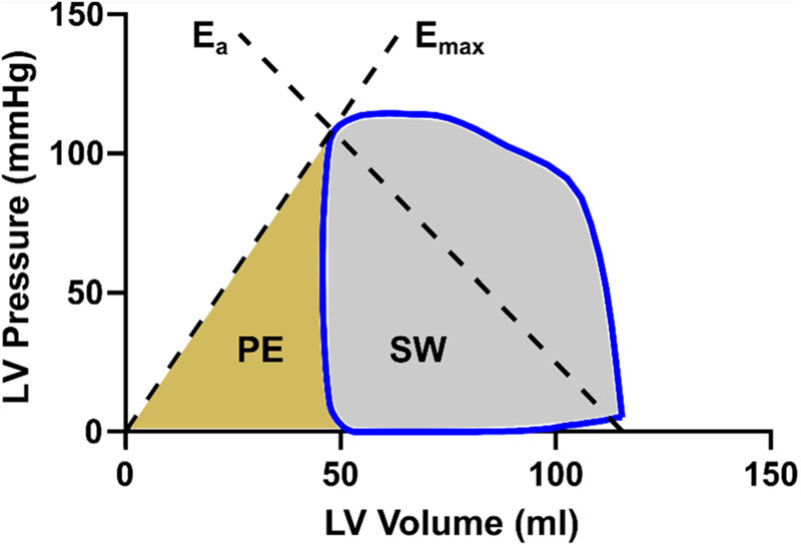
Pressure volume (PV) loop in a healthy volunteer. The diagram shows a loop (blue line) in a healthy volunteer. The variables E_max_, E_a_, PE, and SW have been marked in relation to the PV loop. E_a_, arterial elastance; E_max_, maximal elastance; LV, left ventricular; PE, potential energy; SW, stroke work.

Pressure volume loop variables were defined based on the generated PV loop. Maximal elastance (E_max_, used synonymously with contractility) was defined as the linear slope between the point where the pressure/volume quotient was highest (P_max_) and origo. E_a_ was defined as the linear slope between P_max_ and the point where volume=end‐diastolic volume, and pressure = 0. Stroke work (SW) was defined as the area within the PV loop and PE as the area encapsulated by the E_max_ line, the x‐axis where pressure = 0, and the left side of the PV loop (Figure 1). External power was calculated as the SW normalized to duration of a heartbeat, and energy per volume was calculated as (SW + PE)/stroke volume. Efficiency was calculated as SW/(SW + PE). In addition, the PV‐loop variables were approximated from more conventional functional variables based on end‐diastolic volume, end‐systolic volume, heart rate, and mean arterial pressure (specified in Supporting Information: Figure 1 in the Results section).

#### 2.3.2 Myocardium at risk (MaR) and infarct size (IS)

Regions of MaR were defined by manual delineation of hyperintense regions within the myocardium on the CE‐SSFP short‐axis images in end‐diastole and end‐systole. Size of MaR was expressed as a percentage of the total LV myocardial volume.^16^

IS was assessed from short‐axis LGE images according to a previously described and validated method.^17^ In short, the endocardial and epicardial borders were traced manually excluding the papillary muscles. The LGE myocardium was then quantified by a computer algorithm taking partial volume effects into consideration with manual adjustments when needed to correct for image artifacts.^17^

### 2.4 Statistics

Values are given as mean ± standard deviation unless otherwise specified. The relationships between PV‐loop variables and MaR/IS as well as the relationship between PV‐loop variables and conventional functional variables were assessed using Pearson's correlation coefficient. The agreements between semi‐automatic and manually derived PV‐loop variables were assessed by Bland‐Altman diagrams with bias and limits of agreement. The difference in subject characteristics and PV loop variables between infarct patients and healthy volunteers was assessed using Chi‐square testing. A *p* < .05 was considered to indicate statistical significance. GraphPad version 9.5.0 was used for all statistical analyses.

## 3 RESULTS

A total of 100 patients and 75 healthy volunteers were included in the study. Subject characteristics and conventional functional variables are shown in Table 1. PV loop variables values and comparisons between patients and healthy volunteers are shown in Supporting Information: Table 2.

**Table 1:** Study population characteristics.

Variables	Myocardial infarction	Healthy volunteers	*p* Value
EF, %	48.6 ± 10.0	61.0 ± 5.9	<.001
SV, mL	79.8 ± 18.5	105.9 ± 20.8	<.001
EDV, mL	166.5 ± 34.0	174.3 ± 32.9	.131
ESV, mL	86.8 ± 28.1	68.4 ± 18.4	<.001
SBP, mmHg	124 ± 18	125 ± 13	.680
DBP, mmHg	72 ± 10	75 ± 10	.116
SVR, mmHg×min/L	17.2 ± 5.1	14.4 ± 3.2	<.001
MaR, %LV	32.8 ± 11.2	n/a	n/a
MI size, %LV	17.2 ± 10.6	n/a	n/a
LAD culprit, %	51	n/a	n/a
RCA culprit, %	39	n/a	n/a
LCx culprit, %	9	n/a	n/a
Age, years	62.8 ± 15.6	53.8 ± 12.5	<.001
Sex, % Female	27.7	40.0	.105
Weight, kg	79.1 ± 13.8	76.8 ± 14.2	.748
Height, cm	173.5 ± 9.0	174.7 ± 9.2	.811
Pre‐existing morbidity/medication			
BMI > 30, %	15	9	.660
Hypertension, %	32	n/a	n/a
Diabetes, %	16	n/a	n/a
Smoker/ex‐smoker, %	61	n/a	n/a
ACEi/ARB	21^a^	n/a	n/a
Betablocker	11^a^	n/a	n/a

*Note*: For all characteristics *n* = 100 patients with myocardial infarction and *n* = 75 healthy volunteers. Chi‐square testing was used to calculate *p* values. Values stated as mean ± SD.

Abbreviations: ACEi, angiotensin converting enzyme inhibitor; ARB, angiotensin receptor blocker; BMI, body mass index; DBP,diastolic blood pressure, EDV, end‐diastolic volume; EF, ejection fraction; ESV, end‐systolic volume; LAD, left anterior descending artery; LCx, left coronary circumflex artery; MaR, myocardium at risk; MI, myocardial infarction; RCA, right coronary artery; SV, stroke volume (EDV‐ESV); SVR, systemic vascular resistance; SBP, systolic blood pressure.

^a^ Pre‐existing treatment with ACEi or betablockers was not reported in the CHILL‐MI trial and thus data was missing in 11 patients.

### 3.1 PV‐loop variables versus MaR and IS

**Figure 2:**
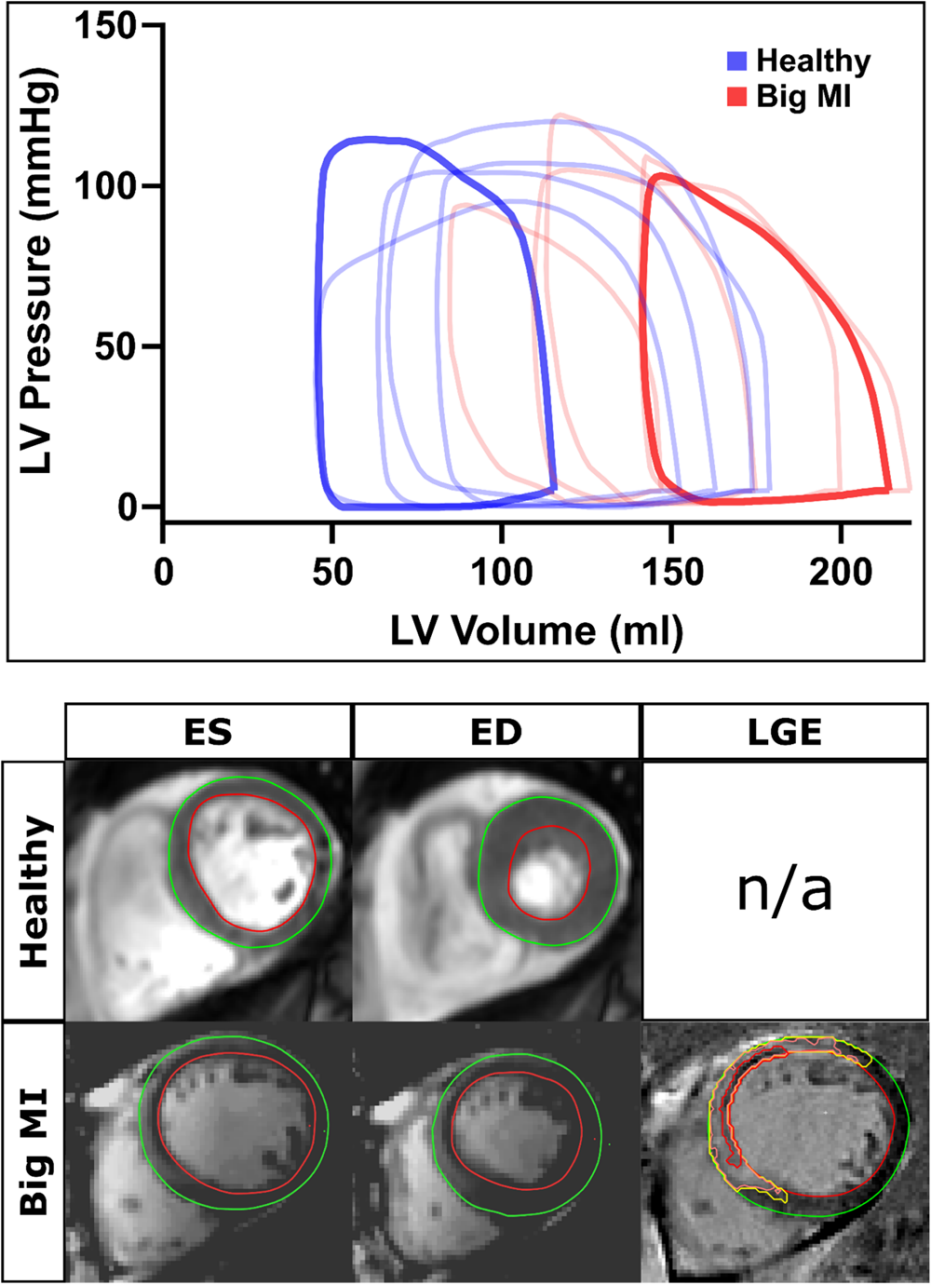
Example PV loops and corresponding CMR images. The diagram shows examples of PV loops from 5 healthy volunteers (blue) and 5 patients after MI (red). The panels below show corresponding MR images for the opaquely colored loops. CINE images are shown in ED and ES (left) and infarct is visualized in the LGE image (right). Green lines indicate epicardium, red lines endocardium, and yellow lines the borders of the infarct zone. The red delineation in the LV myocardium indicates microvascular obstruction. ED, end‐diastole; ES, end‐systole; LGE, late gadolinium enhancement; LV, left ventricle; MI, myocardial infarction.

**Figure 3:**
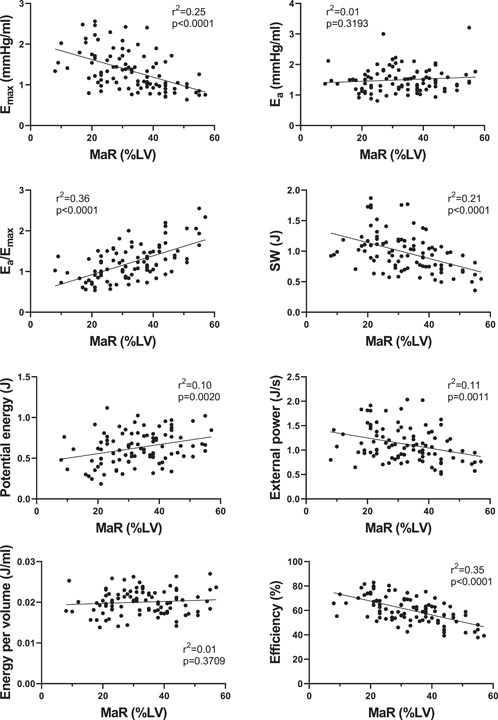
PV loop variables versus myocardium at risk in STEMI patients (*n* = 100 for all graphs). The full line shows linear regression. Pearson's correlation coefficient was used to assess the relationship between the variables and MaR. E_a_, arterial elastance; E_max_, maximal elastance; LV, left ventricle; MaR, myocardium at risk; STEMI, ST‐elevation myocardial infarction; SW, stroke work.

**Figure 4:**
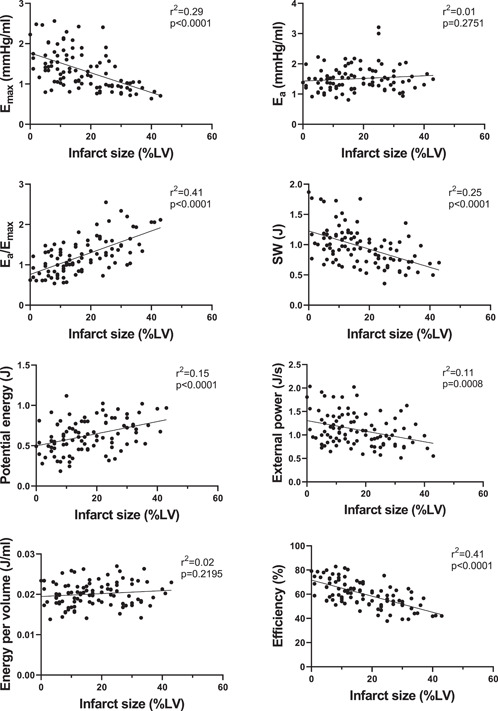
PV loop variables versus infarct size in STEMI patients (*n* = 100 for all graphs). The full line shows linear regression. Pearson's correlation coefficient was used to assess the relationship between the variables and infarct size. E_a_, arterial elastance, E_max_, maximal elastance, LV, left ventricle, STEMI, ST‐elevation myocardial infarction; SW, stroke work.

Figure 2 shows examples of PV loops and CMR images from subjects included in the study. The relationships between PV‐loop variables versus MaR and versus IS are shown in Figures 3 and 4 respectively for all STEMI patients. E_max_, reflecting contractility, was related to both MaR and IS (*r* ^2^ = 0.25, *p* < .001 and *r* ^2^ = 0.29, *p* < .001). Subanalyses of contractility versus MaR and IS for the three separate study populations are shown in Supporting Information: Figure 2. Furthermore, SW, PE, and efficiency were significantly correlated to MaR and IS, whereas E_a_, external power, and energy per volume were not (Figure 3).

### 3.2 PV‐loop variables versus conventional functional variables

Supporting Information: Figure 1 shows the relationship between PV‐loop variables and variables derived from conventional functional measures (end‐diastolic volume, end‐systolic volume, heart rate, and mean arterial pressure). There was a strong correlation with low or no bias for all variables. Supporting Information: Figure 3 shows the relationship between ventriculoarterial coupling and ejection fraction.

### 3.3 Semi‐automatic versus manual PV‐loop variables

The agreements between semi‐automatic spline interpolated PV‐loop variables and manually derived PV‐loop variables are shown in the Supporting Information: Appendix. There was an excellent agreement with low bias and narrow limits of agreement for all variables.

## 4 DISCUSSION

This study is, to the best of our knowledge, the first to apply noninvasive CMR‐based PV‐loop variables in patients early after acute MI. The study concludes that PV‐loop variables such as E_max_ (reflecting contractility), E_a_/E_max_ (reflecting ventriculoarterial coupling), and SW can be used to assess cardiac hemodynamics early after acute MI and these variables provide incremental pathophysiological information over variables based on pressure or volume alone.

### 4.1 Contractility

Contractility (as assessed by maximal elastance, E_max_) was lower in infarct patients compared to volunteers but showed considerable overlap. This overlap is expected considering the wide clinical spectrum of infarct patients ranging from no symptoms to critical heart failure related to varying degree of myocardial injury (IS/MaR). IS and MaR showed similar correlations to E_max_. This was not previously self‐evident as stunning in the entirety of MaR is to be expected early after an ischemic episode and could have resulted in a higher correlation between MaR and E_max_.^18^ In the chronic phase of a MI, however, IS is expected to be more closely related to E_max_, but this remains to be shown.

### 4.2 Ea/Emax

The Ea/Emax ratio, referred to as ventriculo‐arterial coupling, has previously been investigated as a marker of balance between ventricular contractility and afterload, where maximal efficiency has been calculated at Ea/Emax of 0,5 and maximal SW at Ea/Emax of 1.^19^ In the current study, MI patients showed less efficiency than volunteers while being close to maximal SW, when assessed in absolute numbers. This may reflect the homeostatic drive to maintain cardiac output and pressure by maintaining SW at the cost of pump efficiency. Prognostically, Ea/Emax has been shown to have short‐term significance in intensive cardiac care unit patients using a noninvasive echocardiography‐based method described by Chen et al.^20^, ^21^, ^22^ Additionally, Antonini‐Canterin et al. investigated VA‐coupling after MI and found better prognostic value than brain natriuretic peptide for 5‐year mortality, also using the method described by Chen et al.^22^, ^23^ Still, Ea/Emax is yet to be proven useful as a prognostic marker beyond conventional functional variables such as ejection fraction.

### 4.3 SW and efficiency

SW and efficiency were lower in MI patients compared to healthy volunteers and showed a negative correlation to both MaR and IS. These findings are in line with the study by Hamosh et al.^24^ in which SW was shown to be reduced after acute MI in patients with severe symptoms using a method based on myocardial gated scintigraphy. Furthermore, efficiency and myocardial work as estimated using echocardiography have been shown to be decreased in STEMI patients with versus without complications.^25^, ^26^, ^27^ Thus, SW shows promise as a potential prognostic marker after MI. Indeed, Boe et al. has previously used the relationship between MaR and regional dysfunction to show that regional SW derived from echocardiography can be used to identify coronary occlusion in patients with non‐STEMI.^28^ Regional SW was superior to conventional functional variables such as global and regional myocardial strain as well as ejection fraction. Furthermore, global SW by echocardiography has been shown to be superior to global longitudinal strain for identification of patients with significant coronary artery disease.^29^ While data on SW and mortality seems to be lacking in MI patients, low SW has been associated with higher short‐term and long‐term mortality in a mixed population of cardiac intensive care unit patients.

### 4.4 Noninvasive PV‐loop derived by cardiac imaging

Both echocardiography and CMR has recently been used to study cardiac hemodynamics non‐invasively.^4^, ^6^, ^13^, ^30^, ^31^, ^32^, ^33^, ^34^ Echocardiography is widely accessible and the method of choice for assessment of cardiac function in suspected heart failure according to current guidelines.^35^ However, for cardiac volumetric assessment as input for PV loops, CMR has some advantages. First, it is not limited to acoustic windows and it enables full 3D coverage of blood pool dimensions throughout the cardiac cycle without geometrical assumptions. Therefore, CMR is currently considered the reference standard for assessment of global and regional myocardial function and is well suited for noninvasive PV‐loop analysis.^36^ Furthermore, in the context of ischemic heart disease as in the present study, CMR provides possibilities for assessment of additional myocardial features such as MaR and IS.

As can be seen in Supporting Information: Figure 1, most PV‐loop‐specific variables can be estimated based on conventional variables such as blood pressure, EDV, ESV, and heart rate. Supporting Information: Figure 3 shows the direct relationship between ventriculoarterial coupling and ejection fraction.

### 4.5 Study limitations

This study should be interpreted in light of some limitations. Blood pressures used in this study were acquired on the day of imaging. While blood pressures ideally should be acquired continuously during the acquisition of images, previously published data shows good reproducibility of resting blood pressures with minimal within‐subject variation compared to between‐subject variation.^37^ The method used to calculate PV‐loops is dependent on common characteristics of time‐varying‐elastance between different hearts irrespective of pathology as shown by Senzaki et al where the time‐varying elastance in humans was essentially constant independent of pathology.^38^ The rationale is described in the original publication of this method and the model has been verified against invasive measurements.^4^, ^14^ Significant aortic stenosis would void the assumption that brachial blood pressure can be used to estimate aortic valve opening pressure. All patients, however, had an echocardiography and none was reported to have significant aortic stenosis. Data on individual medications was not available in the acute setting. In the period of the clinical trials, however, >90% of these patients received a beta‐blocker, an ACEi or ARB, a statin, and dual antiplatelet therapy. It is therefore reasonable to assume that most patients in this study received the aforementioned medications at the time of inclusion in the trial.

## 5 CONCLUSIONS

Noninvasive PV‐loops provide physiological information beyond conventional diagnostic variables such as ejection fraction and cardiac dimensions early after MI, including measures of contractility, ventriculoarterial coupling, and SW.

## CONFLICTS OF INTEREST STATEMENT

D. N. has received funding from the Region of Scania, Sweden. D. A. has no disclosures. D. E. has received funding from the Region of Scania, Sweden. U. E. has received funding from the Region of Scania, Sweden, and the Swedish Heart‐Lung Foundation. A. K. has received funding from the Region of Scania, Sweden, and the Crafoord Foundation, Sweden, and the Governor Per Westling Memorial Foundation, Sweden. H. A. has received funding from the Regions of Scania, Sweden, the Swedish heart and lung foundation, and the Medical Faculty of Lund University. The remaining authors declare no conflicts of interest.

## Supporting information

Supporting information.

## Data Availability

Data will be made available upon reasonable request.

## References

[1] Roger VL . Epidemiology of heart failure. Circ Res. 2013;113(6):646‐659. 10.1161/CIRCRESAHA.113.300268 23989710 PMC3806290

[2] Heidenreich PA , Bozkurt B , Aguilar D , et al. 2022 AHA/ACC/HFSA guideline for the management of heart failure. J Am Coll Cardiol. 2022;79(17):e263‐e421. 10.1016/j.jacc.2021.12.012 35379503

[3] Otto F . Die grundform des arteriellen pulses. Z Biol. 1899;37:483‐526. 10.1016/0022-2828(90)91459-k 2192068

[4] Seemann F , Arvidsson P , Nordlund D , et al. Noninvasive quantification of Pressure‐Volume loops from brachial pressure and cardiovascular magnetic resonance. Circ Cardiovasc Imag. 2019;12(1):e008493. 10.1161/CIRCIMAGING.118.008493 30630347

[5] Suga H . Ventricular pumping by its Pressure‐Volume coefficient. Jpn J Med Biol Eng. 1969;7(6):406‐415.

[6] Sjöberg P , Liuba P , Arheden H , Heiberg E , Carlsson M . Non‐invasive quantification of pressure–volume loops in patients with fontan circulation. BMC Cardiovasc Disord. 2022;22(1):253. 10.1186/s12872-022-02686-7 35668358 PMC9169380

[7] Berg J , Jablonowski R , Nordlund D , et al. Mild hypothermia attenuates ischaemia/reperfusion injury: insights from serial non‐invasive pressure–volume loops. Cardiovasc Res. 2023;119:2230‐2243. 10.1093/cvr/cvad028 36734080 PMC10578916

[8] MITOCARE Study Group . Rationale and design of the MITOCARE study: a phase II, multicenter, randomized, Double‐Blind, Placebo‐Controlled study to assess the safety and efficacy of TRO40303 for the reduction of reperfusion injury in patients undergoing percutaneous coronary in. Cardiology. 2012;123(4):201‐207. 23202613 10.1159/000342981

[9] Erlinge D , Götberg M , Lang I , et al. Rapid endovascular catheter core cooling combined with cold saline as an adjunct to percutaneous coronary intervention for the treatment of acute myocardial infarction. J Am Coll Cardiol. 2014;63(18):1857‐1865. 10.1016/j.jacc.2013.12.027 24509284

[10] Khoshnood A , Carlsson M , Akbarzadeh M , et al. Effect of oxygen therapy on myocardial salvage in ST elevation myocardial infarction: the randomized SOCCER trial. Eur J Emerg Med. 2018;25:78‐84. 10.1097/MEJ.0000000000000431 27893526

[11] Simonetti OP , Kim RJ , Fieno DS , et al. An improved MR imaging technique for the visualization of myocardial infarction. Radiology. 2001;218(1):215‐223. 11152805 10.1148/radiology.218.1.r01ja50215

[12] Heiberg E , Sjögren J , Ugander M , Carlsson M , Engblom H , Arheden H . Design and validation of segment‐‐freely available software for cardiovascular image analysis. BMC Med Imaging. 2010;10(1):1. 20064248 10.1186/1471-2342-10-1 PMC2822815

[13] Edlund J , Arvidsson PM , Nelsson A , et al. Noninvasive assessment of left ventricular Pressure‐Volume relations: inter‐ and intraobserver variability and assessment across heart failure subtypes. Am J Cardiol. 2022;184:48‐55. 10.1016/j.amjcard.2022.09.001 36192197

[14] Arvidsson PM , Green PG , Watson WD , et al. Non‐invasive left ventricular pressure‐volume loops from cardiovascular magnetic resonance imaging and brachial blood pressure: validation using pressure catheter measurements. Euro Heart J Imag Meth Pract. 2023;1:qyad035. 10.1093/ehjimp/qyad035 PMC10631830 37969333

[15] Oh T , Ogawa K , Nagoshi T , et al. Relationship between haemodynamic indicators and haemogram in patients with heart failure. ESC Heart Failure. 2023;10(2):955‐964. 10.1002/ehf2.14258 36478404 PMC10053360

[16] Sörensson P , Heiberg E , Saleh N , et al. Assessment of myocardium at risk with contrast enhanced steady‐state free precession cine cardiovascular magnetic resonance compared to single‐photon emission computed tomography. J Cardiovasc Magn Reson. 2010;12(1):25. 20433716 10.1186/1532-429X-12-25 PMC2885384

[17] Heiberg E , Ugander M , Engblom H , et al. Automated quantification of myocardial infarction from MR images by accounting for partial volume effects: animal, phantom, and human study. Radiology. 2008;246(2):581‐588. 18055873 10.1148/radiol.2461062164

[18] Heyndrickx GR , Millard RW , McRitchie RJ , Maroko PR , Vatner SF . Regional myocardial functional and electrophysiological alterations after brief coronary artery occlusion in conscious dogs. J Clin Invest. 1975;56(4):978‐985. 10.1172/JCI108178 1159098 PMC301954

[19] Burkhoff D , Sagawa K . Ventricular efficiency predicted by an analytical model. Am J Physiol Regulat Integrat Compar Physiol. 1986;250(6):R1021‐R1027. 10.1152/ajpregu.1986.250.6.R1021 3717375

[20] Trambaiolo P , Bertini P , Borrelli N , et al. Evaluation of ventriculo‐arterial coupling in ST elevation myocardial infarction with left ventricular dysfunction treated with levosimendan. Int J Cardiol. 2019;288:1‐4. 10.1016/j.ijcard.2019.04.052 31056414

[21] Trambaiolo P , Figliuzzi I , Salvati M , et al. Ventriculo‐arterial coupling in the intensive cardiac care unit: A non‐invasive prognostic parameter. Int J Cardiol. 2022;348:85‐89. 10.1016/j.ijcard.2021.12.026 34933063

[22] Chen C‐H , Fetics B , Nevo E , et al. Noninvasive single‐beat determination of left ventricular end‐systolic elastance in humans. J Am Coll Cardiol. 2001;38(7):2028‐2034. 10.1016/S0735-1097(01)01651-5 11738311

[23] Antonini‐Canterin F , Enache R , Popescu BA , et al. Prognostic value of Ventricular‐Arterial coupling and B‐Type natriuretic peptide in patients after myocardial infarction: a Five‐Year Follow‐Up study. J Am Soc Echocardiogr. 2009;22(11):1239‐1245. 10.1016/j.echo.2009.08.009 19783121

[24] Hamosh P , Cohn JN . Left ventricular function in acute myocardial infarction. J Clin Invest. 1971;50(3):523‐533. 10.1172/JCI106521 5101778 PMC291959

[25] Meimoun P , Abdani S , Stracchi V , et al. Usefulness of noninvasive myocardial work to predict left ventricular recovery and acute complications after acute anterior myocardial infarction treated by percutaneous coronary intervention. J Am Soc Echocardiogr. 2020;33(10):1180‐1190. 10.1016/j.echo.2020.07.008 33010853

[26] Lustosa RP , van der Bijl P , El Mahdiui M , et al. Noninvasive myocardial work indices 3 months after ST‐segment elevation myocardial infarction: prevalence and characteristics of patients with postinfarction cardiac remodeling. J Am Soc Echocardiogr. 2020;33(10):1172‐1179. 10.1016/j.echo.2020.05.001 32651125

[27] El Mahdiui M , van der Bijl P , Abou R , Ajmone Marsan N , Delgado V , Bax JJ . Global left ventricular myocardial work efficiency in healthy individuals and patients with cardiovascular disease. J Am Soc Echocardiogr. 2019;32(9):1120‐1127. 10.1016/j.echo.2019.05.002 31279618

[28] Boe E , Russell K , Eek C , et al. Non‐invasive myocardial work index identifies acute coronary occlusion in patients with non‐ST‐segment elevation‐acute coronary syndrome. European Heart J Cardiovas Imag. 2015;16(11):1247‐1255. 10.1093/ehjci/jev078 25851329

[29] Edwards NFA , Scalia GM , Shiino K , et al. Global myocardial work is superior to global longitudinal strain to predict significant coronary artery disease in patients with normal left ventricular function and wall motion. J Am Soc Echocardiogr. 2019;32(8):947‐957. 10.1016/j.echo.2019.02.014 31043359

[30] Chan J , Edwards NFA , Khandheria BK , et al. A new approach to assess myocardial work by non‐invasive left ventricular pressure–strain relations in hypertension and dilated cardiomyopathy. Euro Heart J Cardiovas Imag. 2019;20(1):31‐39. 10.1093/ehjci/jey131 30247622

[31] Argirò A , Rosenblum H , Griffin J , et al. Sex related differences in exercise performance in patients with hypertrophic cardiomyopathy: hemodynamic insights through non‐invasive pressure volume analysis. Int J Cardiol. 2022;351:78‐83. 10.1016/j.ijcard.2021.12.045 34968627

[32] Seemann F , Berg J , Solem K , et al. Quantification of left ventricular contribution to stroke work by longitudinal and radial force‐length loops. J Appl Physiol. 2020;129(4):880‐890. 10.1152/japplphysiol.00198.2020 32816638 PMC8285573

[33] Sjöberg P , Seemann F , Arheden H , Heiberg E . Non‐invasive quantification of pressure‐volume loops from cardiovascular magnetic resonance at rest and during dobutamine stress. Clin Physiol Funct Imaging. 2021;41(5):467‐470. 10.1111/cpf.12718 34121316

[34] Sjöberg P , Arheden H , Heiberg E , Stephensen S , Carlsson M . Haemodynamic left‐ventricular changes during dobutamine stress in patients with atrial septal defect assessed with magnetic resonance imaging‐based pressure–volume loops. Clin Physiol Funct Imaging. 2022;42(6):422‐429. 10.1111/cpf.12781 35838181 PMC9796342

[35] McDonagh TA , Metra M , Adamo M , et al. 2021 ESC guidelines for the diagnosis and treatment of acute and chronic heart failure. Eur Heart J. 2021;42(36):3599‐3726. 10.1093/eurheartj/ehab368 34447992

[36] Hendel RC , Patel MR , Kramer CM , et al. ACCF/ACR/SCCT/SCMR/ASNC/NASCI/SCAI/SIR 2006 appropriateness criteria for cardiac computed tomography and cardiac magnetic resonance imaging. J Am Coll Cardiol. 2006;48(7):1475‐1497. 10.1016/j.jacc.2006.07.003 17010819

[37] Stanforth PR , Gagnon J , Rice T , et al. Reproducibility of resting blood pressure and heart rate measurements. Ann Epidemiol. 2000;10(5):271‐277. 10.1016/S1047-2797(00)00047-8 10942874

[38] Senzaki H , Chen C‐H , Kass DA . Single‐beat estimation of end‐systolic pressure‐volume relation in humans. Circulation. 1996;94(10):2497‐2506. 10.1161/01.CIR.94.10.2497 8921794

