# Supplementary material for: Contractility, ventriculoarterial coupling, and stroke work after acute myocardial infarction using CMR‐derived pressure‐volume loop data
Source: Clin Cardiol. 2024 Jan 16;47(1):e24216. doi: 10.1002/clc.24216 (PMC10790509; doi:10.1002/clc.24216)
Supplement: Supplementary file 1 — Supporting information. [file CLC-47-e24216-s001.docx]

**Supplemental Appendix**

**-** Supplemental validation results (referenced in methods), page 2-3

- Supplemental Tables, page 3-4

- Supplemental Figure Legends, page 5

- Supplemental Figures, page 6-7

**Supplemental results**

**
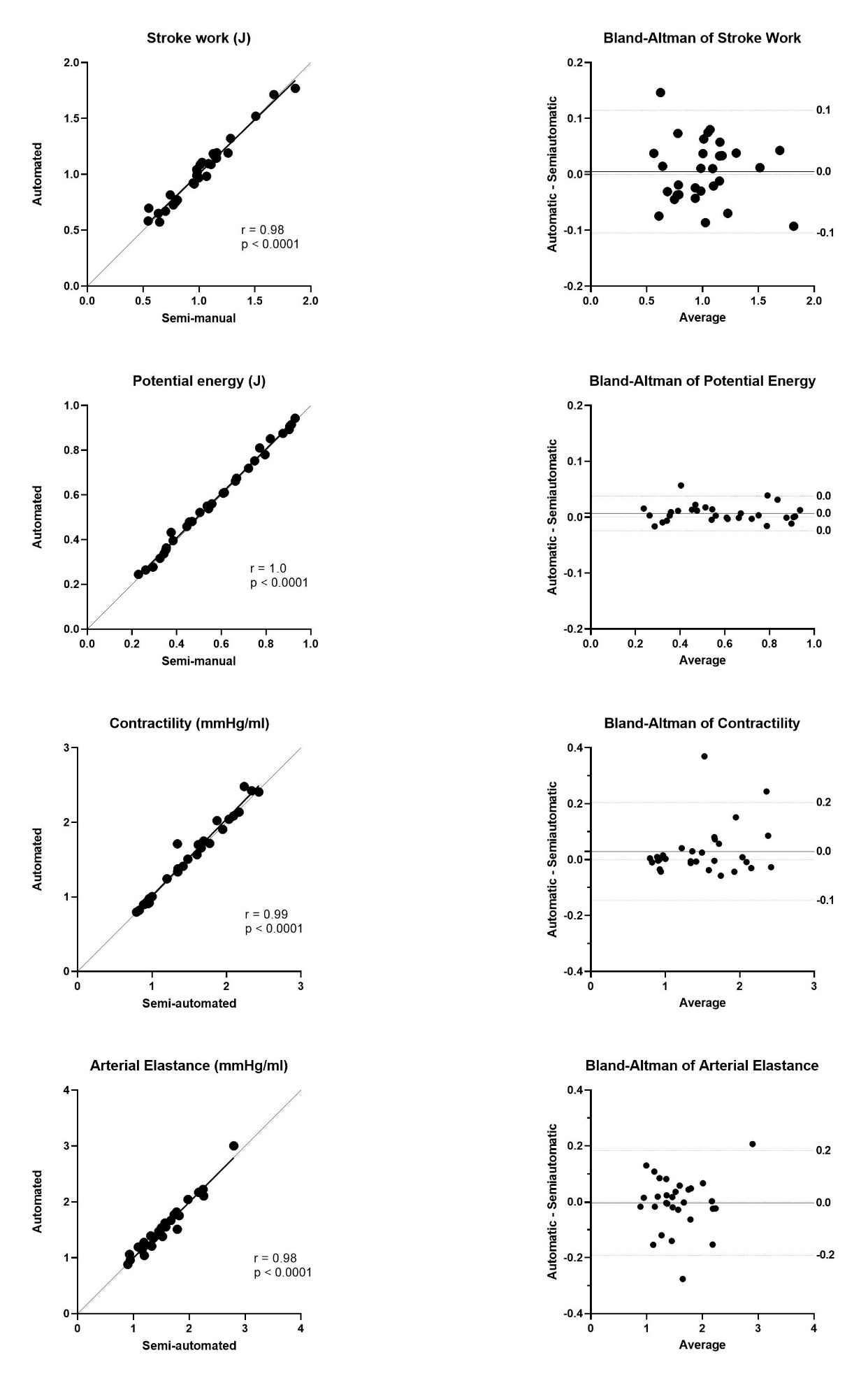
**

**Semi-automatic vs manual PV-loop delineations.** The graphs show the correlations between a semi-automatic method of delineating time-resolved left ventricular volumes and fully manual delineations. The correlation plots to the left show lines of identity as broken lines and linear regression lines as full lines. The Bland-Altmann graphs to the right show mean as full lines and ±2SD as broken lines.

**Supplemental tables**

**Supplemental Table 1.**

Comparison of inclusion and exclusion criteria for the SOCCER, CHILL-MI, and Mitocare trials.

| **SOCCER** | **CHILL-MI** | **Mitocare** |
| --- | --- | --- |
| ST-elevation myocardial infarction | ST-elevation myocardial infarction | ST-elevation myocardial infarction |
| Symptom duration <6 h | Symptom duration <6 h | Symptom duration <6 h |
| No prior myocardial infarction | No prior myocardial infarction | No prior myocardial infarction |
| Able to undergo CMR scanning | Able to undergo CMR scanning | Able to undergo CMR scanning |
| SaO_2_ > 93% | Persistent ST-elevation on a second ECG obtained in the catheterization laboratory with strict criteria for defining ST-elevation (see reference for details) | Persistent occlusion of culprit artery with TIMI flow 0-1 prior to PCI |
| - | No prior cardiac arrest, PCI, CABG, congestive heart failure, end-stage kidney disease, hepatic failure, recent stroke, coagulopathy, pregnancy | No prior CABG, angina within 48h before infarction, iv fibrinolytic therapy within 72h og PCI, atrial fibrillation, pacemaker, inflammatory/infectious/malignant disease, biliary obstruction, hepatic failure |
| - | 18-79 years old | >18 years old |

Only criteria deemed relevant have been included in this table, for an exhaustive description please see the original publications (7–9). CMR=cardiovascular magnetic resonance, ECG=electrocardiogram, PCI=percutaneous coronary intervention, CABG=coronary artery bypass graft

**Supplemental Table 2.** Pressure-volume loop variables.

| **Variables** | **Myocardial infarction** | **Healthy volunteers** | **p-value** |
| --- | --- | --- | --- |
| E_max_, mmHg/ml | 1.34±0.48 | 1.50±0.41 | 0.024 |
| E_a_, mmHg/ml | 1.52±0.47 | 1.06±0.28 | <0.001 |
| E_a_/E_max_ | 1.27±0.61 | 0.73±0.17 | <0.001 |
| SW, J | 0.96±0.32 | 1.38±0.32 | <0.001 |
| PE, J | 0.63±0.22 | 0.47±0.15 | <0.001 |
| External power, J/s | 1.11±0.36 | 1.46±0.35 | <0.001 |
| Energy per volume, J/ml | 0.020±0.004 | 0.018±0.002 | <0.001 |
| Efficiency, % | 59.9±11.8 | 74.5±6.1 | <0.001 |

For all variables n=100 patients with myocardial infarction and n=75 healthy volunteers. Chi-square testing was used to calculate p-values. E_a_=arterial elastance, E_max_=maximal elastance, PE=potential energy, SW=stroke work.

**Supplemental Figure legends**

**Supplemental Figure 1. PV loop variables** **correlated to conventional variables (n=175 for all graphs).** Linear regression is shown as the full line and the broken line is the line of identity. These illustrations show the variables derived by PV loops on the Y-axes and the same variables estimated by calculations based on traditional variables (EDV, ESV, MAP, heart rate). Note the high degree of agreement which validates the robustness of the time-varying elastance model of generating PV loops. Pearson’s correlation coefficient was used to assess the relationship between the variables. EDV=end-diastolic volume, ESV=end-systolic volume, MAP=mean arterial pressure, PV=pressure-volume.

**Supplemental Figure 2. Separate analyses of E_max_ (contractility) vs infarct size and MaR in the CHILL-MI, MITOCARE, and SOCCER trial patients.** Linear regression is shown as the full line. Note the higher correlation between E_max_ and MaR compared to infarct size in the CHILL-MI population which may have had stricter inclusion criteria (c.f. Supplemental Table 1). Pearson’s correlation coefficient was used to assess the relationship between the variables and infarct size/MaR. E_max_=maximal elastance, LV=left ventricle, MaR=myocardium at risk

**Supplemental Figure 3. Ventriculoarterial coupling (VAC) vs ejection fraction (EF), n=175.** Note the close agreement between VAC and EF which is expected as VAC (calculated as Ea/Emax) directly relates to 1/EF-1 (see Supplemental Figure 2). Pearson’s correlation coefficient was used to assess the relationship between VAC and EF. VAC=ventriculoarterial coupling, EF=ejection fraction, Ea=arterial elastance, Emax=maximal elastance.

**Supplemental Figures**

**Supplemental Figure 1.**

**Supplemental Figure 2.**

**Supplemental Figure 3.**
